# Preparation and In Vitro Characterization of Alkyl Polyglucoside-Based Microemulsion for Topical Administration of Curcumin

**DOI:** 10.3390/pharmaceutics15051420

**Published:** 2023-05-06

**Authors:** Cristina Scomoroscenco, Mircea Teodorescu, Cristina Lavinia Nistor, Ioana Catalina Gifu, Cristian Petcu, Daniel Dumitru Banciu, Adela Banciu, Ludmila Otilia Cinteza

**Affiliations:** 1 Polymer Department, National Institute for Research and Development in Chemistry and Petrochemistry-ICECHIM, 202 Spl. Independentei, 060021 Bucharest, Romania; 2 Faculty of Applied Chemistry and Materials Science, University Politehnica of Bucharest, 1-7 Gh. Polizu Street, 011061 Bucharest, Romania; 3 Department of Biomaterials and Medical Devices, Faculty of Medical Engineering, Politehnica University of Bucharest, 1-7 Gh. Polizu Street, 011061 Bucharest, Romania; 4 Physical Chemistry Department, University of Bucharest, 4-12 Blv. Regina Elisabeta, 030018 Bucharest, Romania

**Keywords:** topical formulations, curcumin delivery, gel microemulsion, transdermal delivery, microemulsion, skin penetration

## Abstract

The skin is a complex and selective system from the perspective of permeability to substances from the external environment. Microemulsion systems have demonstrated a high performance in encapsulating, protecting and transporting active substances through the skin. Due to the low viscosity of microemulsion systems and the importance of a texture that is easy to apply in the cosmetic and pharmaceutical fields, gel microemulsions are increasingly gaining more interest. The aim of this study was to develop new microemulsion systems for topical use; to identify a suitable water-soluble polymer in order to obtain gel microemulsions; and to study the efficacy of the developed microemulsion and gel microemulsion systems in the delivery of a model active ingredient, namely curcumin, into the skin. A pseudo-ternary phase diagram was developed using AKYPO^®^ SOFT 100 BVC, PLANTACARE^®^ 2000 UP Solution and ethanol as a surfactant mix; caprylic/capric triglycerides, obtained from coconut oil, as the oily phase; and distilled water. To obtain gel microemulsions, sodium hyaluronate salt was used. All these ingredients are safe for the skin and are biodegradable. The selected microemulsions and gel microemulsions were physicochemically characterized by means of dynamic light scattering, electrical conductivity, polarized microscopy and rheometric measurements. To evaluate the efficiency of the selected microemulsion and gel microemulsion to deliver the encapsulated curcumin, an in vitro permeation study was performed.

## 1. Introduction

Curcumin (CURC), the polyphenolic main component of the Turmeric spice, has been recognized for its potential in the treatment of many diseases, and it has been used in traditional medicine for a long time. In the last two decades, it has been extensively investigated in order to evidence its mechanism of action as an antimicrobial and antitumoral agent and to elucidate its beneficial role in diabetes, cardiovascular and gastrointestinal diseases, degenerative diseases (such as Parkinson’s, and Alzheimer’s), etc., resulting in promising results, even in clinical trials [1].

One of the most interesting and valuable properties of the curcuminoid compounds found in Curcuma powder, and of curcumin in particular, is their antioxidant activity, which has received much interest in both the medical and cosmetic fields. Recent studies have revealed the different mechanisms involved in the antioxidant activity of curcumin, such as the scavenging of various reactive oxygen and nitrogen species and the inhibition of ROS-generating enzymes [2].

Complementary to the development of topical products with curcumin for dermatological purposes, the scientific evidence of its antioxidant and anti-inflammatory efficiency has led to its use as a cosmetic active ingredient. For example, the ability of CURC to block signaling pathways dependent on PhK results in the mitigation of tissue injuries and supports anti-aging effects [3]. Furthermore, among the natural polyphenol compounds, curcumin has been proven to possess a superior ability to protect the skin against the harmful effects from exposure to UV radiation. An evaluation of the SPF performance and hazardous risks of CURC compared with those of commercially available sunscreen products concluded that curcumin, together with resveratrol and ferulic acid, could be considered a safer viable alternative to cosmetic creams formulated with conventional chemical UV absorbers with a high SPF [4].

However, sensitive phytochemicals, such as CURC, need to be encapsulated in suitable carriers in order to improve their stability, solubility and skin delivery properties. The high hydrophobicity of curcumin molecules represents a disadvantage in topical delivery [5], and strategies to enhance permeation have been proposed by using permeation enhancers or various nanocarriers [6]. Thus, the encapsulation of curcumin in various formulations designed for topical administration has been studied, and promising results have been reported with nanoemulsions [7], liposomes [8], cubosomal hydrogels, transferosomes in gels [9], cosmetic creams with liposomes and ethosomes [10].

Among the nanostructured colloidal systems proposed as drug delivery systems in topical applications, microemulsions (MEs) are considered to be excellent vehicles, showing a plethora of advantages related to the small dimension of the liquid droplets, optical isotropy and thermodynamic stability. Microemulsions are able to increase the solubilization of a wide range of drugs, as well as protecting entrapped active pharmaceutical ingredients from degradation, hydrolysis and oxidation. They can also offer sustained release, resulting in a reduction in the side effects of problematic drugs with a high toxicity [11]. In particular, in topical delivery, MEs have been found to decrease the irritation of some active substances compared to their solutions [12]. Oil–water ratios have recently been discovered to be critical for colloidal stability and transdermal drug administration in numerous microemulsion systems [13,14]. Various curcumin-encapsulating microemulsions, optimized for specific therapeutic tasks or administration routes, have been reported. For example, a microemulsion formulated with curcumin, turmeric oil and a mixture of Kolliphor RH40–ethanol as a surfactant–cosurfactant pair exhibited a protective effect against induced neurodegeneration in zebrafish, as reported by More et al. [15]. Amuti et al. reported a microemulsion with Cremophor EL/ethanol as a surfactant mixture and isoamyl acetate oily phase that shows superior antioxidant stability [16]. In another previous study, the transdermal delivery of curcumin encapsulated in a microemulsion obtained from water, isopropyl palmitate, Labrasol^®^ and glyceryl oleate as surfactants and propylene carbonate as a cosurfactant was significantly increased and depended on the water content [17].

Besides the obvious benefits for the stability and release kinetics of CURC, the current formulations lack some critical qualities, such as the use of pharmaceutically/cosmetically accepted ingredients and a high solubilization capacity [18,19]. Regarding the oily phase of microemulsions, the most challenging are vegetable oils and some triglycerides from vegetable oils, particularly because of the formation of undesirable phases, such as liquid crystals and macroemulsions [20]. The difficulties in the microemulsification of natural oils are reflected in the reduced number of papers that report the successful preparation of single-phase microemulsions where the oily phase consists of vegetable oils [21,22,23,24] or triglycerides [20,25,26].

Because vegetable oils for cosmetic applications are highly appreciated, can simultaneously act as an active ingredient with an emollient and have regenerating properties for the skin, in this study, caprylic/capric triglycerides (CCTs) obtained from coconut oil were used as the oily phase. They are a blend of medium-chain fatty acids, with the major part consisting of a caprylic and capric acid blend (C8–C10 fatty acids) [27], and they are widely used in the cosmetic industry and are also appreciated due to their reduced ecological impact.

The major challenge in the formulation of a microemulsion for dermatocosmetic use is the selection of a surfactant mixture. In the present study, a mixture of surfactants (S_mix_) consisting of AKYPO^®^ SOFT 100 BVC (Akypo), Plantacare^®^ 2000 UP Solution (Plantacare) and ethanol was used to ensure the microemulsion formation. Akypo is an environmentally friendly anionic surfactant and is non-irritating for the skin, with an ethoxy chain length equal to 10 [28]. Akypo was selected as a component of S_mix_ specifically for its ethoxy chain, which confers more flexibility to the surfactant film, an important parameter for microemulsion formation [20]. Plantacare is an alkylpolyglucoside, non-ionic surfactant that is 100% renewable and sustainable, with a good dermatological compatibility [29].

Unfortunately, because of a microemulsion’s low viscosity, it is quickly removed from the skin and is difficult to apply as a home cosmetic product. Thickening agents are used to increase its viscosity, leading to the nanostructured carrier currently known as a gel microemulsion [30]. Based on our previous work, a high-molecular-weight sodium hyaluronate salt (NaH) was used to obtain gel microemulsions [24], as hyaluronic compounds are frequently used in dermatocosmetics due to their intrinsic hydrating and regenerative properties.

The aim of this study was to develop a new gel microemulsion system with increased penetration properties for the topical delivery of curcumin using nontoxic renewable surfactants and a polymer. Surfactants Akypo and Plantacare have been chosen since they are ingredients that are already approved for dermatocosmetic formulations and many papers have reported reduced cytotoxicity on a great number of normal mammalian cells and very low toxic effect on the epidermal and dermal layers, compared to other nonionic surfactants [31,32,33].

To the best of our knowledge, this is the first time the surfactants Akypo and Plantacare were studied as a pair to create a microemulsion where the oily phase is composed of CCTs.

## 2. Materials and Methods

### 2.1. Materials

Caprylic/capric triglycerides (MAYAM Cosmetics, Oradea, Romania); AKYPO^®^ SOFT 100 BVC (Sodium Laureth-11 Carboxylate was kindly gifted by Kao Chemicals Europe, Barcelona, Spain), ethanol (>99.8%) and curcumin (>99%) (Sigma-Adrich Chemie GmbH, Taufkirchen, Germany); sodium hyaluronate with 1–1.6 mDa (MAYAM Cosmetics, Oradea, Romania), DPPH (2,2-diphenyl-1-picrylhydrazyl, Sigma-Aldrich) and Strat-M^®^ membrane (EMD Millipore, Burlington, MA, USA) were used as received. PLANTACARE^®^ 2000 UP Solution (Decyl glucoside (C8–C16), Fluka Chemie GmbH, Buchs, Switzerland) was lyophilized before use. For all experiments, distilled water was used. Phosphate-buffered saline 10× solution (Fluka Chemie GmbH, Buchs, Switzerland) was used after 10-fold dilution with distilled water.

### 2.2. Pseudo-Ternary Phase Diagram Construction

A pseudo-ternary phase diagram was constructed using the water phase titration method. Firstly, the oily phase (CCTs) was mixed with the selected surfactant mixture; secondly, titration with water was performed.

The mass ratio between Plantacare and Akypo was fixed at 2:1. The surfactant mixture, formed from three elements, consisted of 88%wt.% Akypo with Plantacare and 12 wt.% of ethanol. In the first stage, the S_mix_ described above was mixed with the oily phase at 90:10, 85:15, 80:20, 75:25, 70:30, 65:35, 55:45, 45:55, 35:65, 25:75 and 15:85 weight ratios. In the second stage, the water phase was added in small portions to each S_mix_ with the CCT mixture at room temperature, mixing the composition properly and leaving the sample until complete equilibration. Each sample obtained as a result of titration with water was observed, and the type of system that was obtained was noted. This process was repeated until a macroemulsion layer was obtained, as this means that the microemulsion area was exceeded.

Because commercially available Plantacare (PC) is a water solution, an extra step was carried out in order to remove the water. Therefore, PC was lyophilized using a laboratory freeze dryer ALPHA 1-2 LD plus (Martin Christ, Osterode am Harz, Germany). To prevent the absorption of water, the lyophilized PC was stored in a desiccator.

For cosmetic and pharmaceutical applications, single-phase-type microemulsions, that is, Winsor IV (W IV) microemulsions, are of interest. Therefore, the systems were classified as Winsor IV microemulsions when they appeared to be one-phase clear liquids without an additional separated phase. The formation of Winsor I-, II- and III-type microemulsions was also monitored and noted to define the entire microemulsion area [34].

The data obtained after titration of the sample were gathered to construct a pseudo-ternary phase diagram. The software TriPlot version 4.1 was used.

### 2.3. Characterization of Winsor IV Microemulsions

A primary visual observation of the formation of Winsor IV microemulsions was noted. Moreover, the W IV microemulsions were scanned using cross-polarized light microscopy (Optica Microscopes Instruments, Ponteranica, Italy); the images were produced with a set-up Microscope video camera (Levenhuk M1400 PLUS, Tampa, FL, USA). A small drop of microemulsion was placed on a microscope slide and covered with a plastic cover slip. Pictures were taken at 10× magnification at room temperature. Using dynamic light scattering (DLS, Nano ZS ZEN3600 Zetasizer, Malvern Instruments, Malvern, UK) equipment, the microemulsion droplet size was determined. On undiluted samples, electrical conductivity was measured in triplicate using a Cole-Parmer 500 (Cole-Parmer Instrument Co. Europe, St. Neots, UK) conductivity meter. The dynamic viscosity of the samples was measured using a Kinexus Pro rheometer (Malvern Instruments, Malvern, UK), equipped with a Peltier element for temperature control, with 1.60 software for data acquisition. The measurements were performed at room temperature (25 °C) in rotation mode using a 1°/40 mm cone. Before the measurements, an equilibration time of 5 min was set for each sample. The FTIR spectra were collected using a Vertex 70 equipment (Bruker Optics, Ettlingen, Germany) in the ATR mode, in the range of 400–4000 cm^−1^. The small quantities of sample (pure component as powders and microemulsion-based formulation as liquids) were placed on the diamond crystal of the ATR module and analyzed. OPUS software package was used for normalization and baseline correction.

### 2.4. Preparation of Gel Microemulsions

Gel microemulsions were prepared by the addition of a hydrophilic polymer with gelling properties. The selection of the polymer was made to match the cosmetic or pharmaceutic application. Two concentrations of the gelling agent were selected to obtain the desired viscosity and to analyze how increasing the polymer concentration influences the viscosity. It was also taken into account that a polymer with a concentration that is too high will not be completely solubilized in the microemulsion systems. Two W IV MEs were selected as a base for the gel microemulsions, one with a low concentration of the aqueous phase and one with a high concentration of the aqueous phase, namely, the formulations F1.1 and F1.4. As a gelling agent, a hyaluronic acid Na salt of 1–1.6 mDa (NaH) was selected for its capacity to form a gel at low concentrations and its important benefits for the skin as an active ingredient. Two NaH addition methods were evaluated. In the first method, NaH was added to the already prepared microemulsion, followed by magnetic stirring for 24–48 h until the complete dissolution of the polymer. In the second method, a gel microemulsion was obtained in two stages: stage 1 involved the solubilization of the polymer in the aqueous phase of the microemulsion composition, using magnetic stirring, and stage 2 involved the addition of the rest of the components of the microemulsion system. During the addition of the thickening agent, the ratios of the microemulsion components were kept constant.

### 2.5. Characterization of Gel Microemulsions

A primary visual observation of the formation of the gel microemulsions was noted. The complete dissolution of the polymer and the maintained transparency and homogeneity of the microemulsion confirmed the formation of the gel microemulsion in the first step. As the main purpose was to increase the viscosity, the modification of this parameter was monitored using rheological measurements. The dynamic viscosity of the samples was measured with a Kinexus Pro rheometer (Malvern Instruments, Malvern, UK), as described for the parent microemulsions in Section 2.3. FTIR analysis was also performed as described earlier for microemulsions.

### 2.6. Loading Capacity of the Microemulsion and Gel Microemulsions

Some preliminary steps were performed to prepare the samples for a system capacity evaluation in order to incorporate curcumin. An excess of curcumin was added to the samples, followed by magnetic stirring for 48 h to ensure the complete dissolution of the active ingredient. To separate the excess curcumin, the samples were centrifugated for 40 min at 20,000 rpm. The loading capacity of the curcumin in the microemulsion and the gel microemulsion was evaluated using a spectrofluorimetric method adapted from the literature [35] in our laboratory and previously reported [36]. The quantification of CURC was performed using the fluorescence spectra of the samples diluted in ethanol and recorded using a spectrofluorometer Jasco FP6300 (Jasco Corporation, Tokyo, Japan). Excitation was performed at λ_ex_ = 425 nm, and the fluorescence emission was recorded in the range of 450–700 nm. Calibration curves were determined for the fluorescence maximum emission at 525 nm for CURC in ethanol, in the presence and absence of the microemulsion components.

### 2.7. In Vitro Antioxidant Activity

The antioxidant activity of CURC encapsulated in various microemulsion-based systems was determined via the reduction of DPPH free radicals, using a method from the literature [37] and modified in our laboratory [38]. Briefly, 10 μL of microemulsion was added to 2.5 mL of DPPH solution with a concentration of 40 mg/L and vigorously stirred to ensure the homogeneity of the samples. The mixture was incubated in the dark for 15 min (the period was optimized in our previous work for experiments performed with viscous gel microemulsions), and the absorbance was recorded at 517 nm using a UV-VIS spectrophotometer Jasco V-530 (Jasco, Japan).

Curcumin in an ethanolic solution was used as a reference. The antioxidant activity is expressed as the percentage of reduced DPPH free radicals computed using Equation (1):

(1) $$\%\;{\mathrm{DPPH}}_{\mathrm{reduced}}=\frac{A_{0}-A_{s}}{A_{0}}\times 100$$

where A_0_ is the initial absorbance of the DPPH solution without CURC (negative control) and A_s_ is the absorbance of the DPPH solution with the antioxidant (CURC in various media).

### 2.8. In Vitro Drug Release

The release kinetics of curcumin from the selected microemulsion and gel microemulsion was analyzed using the dialysis method. A 12.000–14.000 Dalton MWCO regenerated cellulose dialysis membrane with tubing of 6.4 mm diameter Spectra/Por™ (Spectrum™, New Brunswick, NJ, USA) was used, after being hydrated for 24 h before the experiment. The dialysis membrane was filled with a weighted amount of sample and fully submerged in the receptor medium that surrounds the sample, ensuring the absence of air bubbles, under mild magnetic stirring at a speed of 200 rpm. A 1:1 (*v*/*v*) water/ethanol mixture was used as the receptor media. A constant volume was maintained by regularly drawing out 1 mL samples of the receptor medium and replacing them with fresh receptor medium. The quantification of curcumin in the samples was performed based on a spectrofluorimetric method adapted from the literature and reported in our previous paper [36] The maximum fluorescence emission was measured at 525 nm (excitation wavelength set at 425 nm). The experiments were carried out in triplicate at 25 °C. The results were expressed as the percentage of total drug released from the total amount encapsulated [17]. The release mechanism of curcumin from the microemulsion and gel microemulsion was investigated by fitting the release curves with the following models described in the literature: First-order model, Higuchi’s model, Korsmeyer–Peppas, Peppas–Sahlin and Weibull [39,40]. The mathematic interpretation of experimental data was performed using the DDSolver^®^ software program [41].

### 2.9. In Vitro Permeation Study

A Franz diffusion cell (PermeGear, Inc., Hellertown, PA, USA) with an effective diffusion area of 0.99 cm^2^ was used to perform an in vitro skin permeation study. This technique was used to determine the release rate of curcumin from the microemulsion and gel microemulsion systems. In order to perform accurate dermal permeation, a synthetic membrane with structural and chemical characteristics similar to those of human skin, namely, the Strat-M^®^ membrane, was selected. This membrane does not require prior hydration or any other pretreatment [42]. The Strat-M^®^ membrane was placed with the shiny part exposed to the donor compartment. The receptor compartment with an 8 mL capacity was filled with a 50% (*w*/*w*) solution of ethanol in PBS at pH 7.4 [34,43]. The receptor solution was constantly stirred using a magnetic bar at 300 rpm and maintained at 37 °C. The formulations loaded with curcumin (0.5 g) were placed in the donor compartment, and this was sealed with Parafilm^®^ to prevent sample evaporation during the experiment. At each time point of 2, 3, 4, 5, 6, 12 and 24 h time intervals, 1 mL of sample was removed from the receptor and kept for further analysis, followed by an immediate replacement with an equal volume of a fresh receptor solution. Moreover, the retained curcumin in the synthetic membrane was analyzed. At the end of each experiment, the membrane was cleaned of excess sample. The part exposed to the samples was cropped, cut into small pieces, soaked in 5 mL of ethanol and stirred using a magnetic bar. In order to obtain statistically significant data, all experiments were performed in triplicate. The samples were analyzed using a spectrofluorometer under the same conditions as described in the previous section. The results were plotted as cumulative curcumin permeation versus time. A standard calibration curve was produced using samples with seven concentrations in a range of 0.05–1 μg/mL curcumin in 50% ethanol solution, with a correlation coefficient R^2^ = 0.9992.

The obtained data were used to determine the cumulative curcumin permeation, Qt (μg cm^−2^), of the microemulsion and the gel microemulsion per unit of surface area, the steady-state permeation flux, Js (μg cm^−2^ h^−1^), and the apparent permeability coefficient, Pap (cm h^−1^), calculated using equations from the literature [43].

### 2.10. Confocal Microscopy and MTT Test

The cytotoxicity of the optimized gel microemulsions was evaluated using a viability test based on the MTT method and a microscopy image analysis. L929 mouse fibroblasts (ATCC) were used to evaluate the cytotoxicity of the investigated samples using MTT and LIVE/DEAD assays. For the MTT (3-(4,5-dimethylthiazol-2-yl)-2,5-diphenyltetrazolium bromide) (Sigma, M2128) evaluation, the cells were cultivated in 96-well tissue culture plates (VWR, Radnor, PA, USA) at a density of 3 × 10^5^ cells/mL and maintained in DMEM (Biowest, Nuaillé, France), 10% FBS (Biowest, Nuaillé, France) and 1% penicillin–streptomycin (Sigma) for 24 ± 2 h at 37 °C, 5% CO_2_. After incubation, the medium was removed, and the samples at different concentrations (1%, 0.1%, 0.01%, 0.001% and 0.0001%) were added for 24 h. The cells were incubated for 4 h with 1 mg/mL MTT, and after DMSO membrane solubilization, the absorbance was measured at 570 nm and 690 nm using a DS-11 FX+ Spectrophotometer (DeNovix, Wilmington, DE, USA).

Confocal microscopy was used for the imaging of the cells after exposure to different concentrations of the CURC-loaded gel microemulsion proposed for topical administration. L929 mouse fibroblasts (ATCC) were used as model cells to evaluate the interaction and cytotoxicity of the investigated samples, based on the LIVE/DEAD assays, using calcein AM (LIVE/DEAD Viability/Cytotoxicity Kit for mammalian cells, Life Technologies, Carlsbad, CA, USA) and propidium iodide (PI) (Sigma Aldrich, Merck Group, Darmstadt, Germany). The cells were seeded at an initial concentration of 1 × 10^5^ cells/mL in 24-well plates, and then maintained for 24 h in a culture medium and incubated for 48 h with the gel microemulsion samples (F1.4 as a reference formulation without the drug and F1.4NH1 with encapsulated CURC). The cell cultures after incubation with the treatment were maintained for 30 min at room temperature with 2 µM calcein AM and 1 µg/mL propidium iodide. Viability was assessed using confocal fluorescence microscopy on a Zeiss LSM 880 system (Carl Zeiss AG, Jena, Germany) with 488 and 514 nm lasers. The acquired images were processed using ZEN 2.3 software (Zeiss, Jena, Germany).

For the investigation of the internalization of the CURC loaded in the gel microemulsion, the cells were treated with the 458 nm laser, and the fluorescence was measured in the range of 470–570 nm with the confocal fluorescence system.

## 3. Results

### 3.1. Pseudo-Ternary Phase Diagram

**Figure 1:**
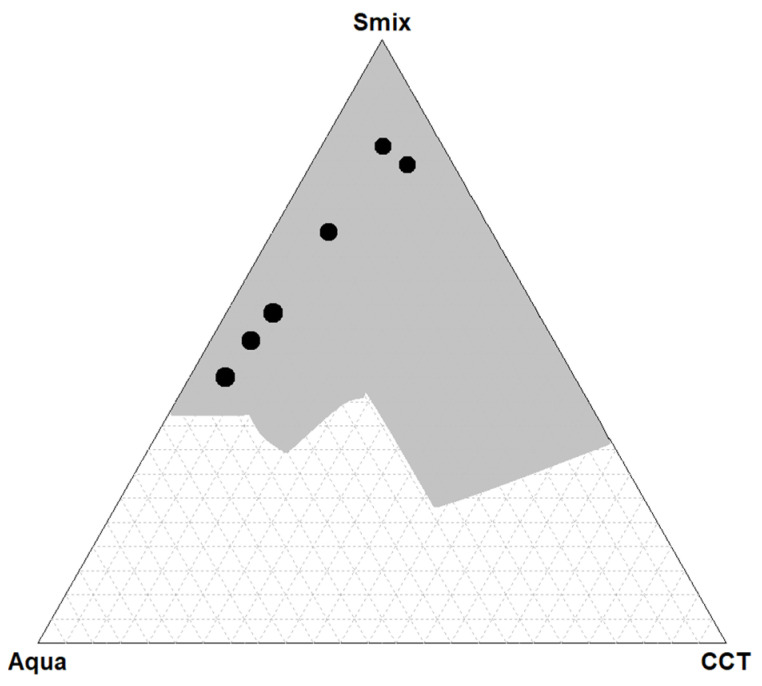
Pseudo-ternary phase diagram of CCTs (oil), surfactant mixture (S_mix_) and water. S_mix_ is a mixture of three components: Plantacare, Akypo and ethanol. The microemulsion area is the gray shaded area. The bullets depict the Winsor IV-type microemulsions.

A pseudo-ternary phase diagram was developed by classifying each titrated sample. Ethanol was included in S_mix_ because it helps to form a microemulsion system, even if vegetable oil or a vegetable oil component is used as the oily phase [43]. Using CCTs as the oily phase and the S_mix_ described above in the Materials and Methods Section, an extended microemulsion area was obtained. The resultant microemulsion area is presented in gray in Figure 1. This area includes all the types of microemulsions obtained, namely, Winsor I and Winsor IV. In Figure 1, it can be seen that a stable microemulsion was formed when the content of S_mix_ was higher than 27% (*w*/*w*).

Because Winsor IV-type microemulsions are of interest, black bullets were used to depict the titrations for which a Winsor IV microemulsion resulted. The formulation entitled F2.1 was from the second dilution line, and the formulations entitled F1.1, F1.2, F1.3, F1.4 and F1.5 were from the first dilution line. Therefore, six Winsor IV microemulsions were obtained in the first and second dilution lines, namely, for 90:10 and 85:15 weight ratios of S_mix_:CCTs. The compositions of the W IV microemulsions are listed in Table 1.

In Table 1 it can be seen that the minimum concentration of S_mix_ required to form a microemulsion in this study was 43.9%.

Increased concentration of the surfactant mixture, beyond 80%, allow the formation of single-phase microemulsions with a relatively high content of the oily phase, at both 90:10 and 85:15 surfactant–cosurfactant molar ratios. However, at the Plantacare–Akypo molar ratio of 80:15, a Winsor IV microemulsion with high water content could also be prepared. Other tested surfactant–cosurfactant molar ratios did not produced any single-phase microemulsions, even at the highest surfactant concentrations.

### 3.2. Physical Characterization of Winsor IV Microemulsions

Due to the fact that a Winsor IV microemulsion is a transparent one-phase system, it is easy to differentiate it from Winsor I, II and III microemulsions or non-microemulsion systems. The compositions of the single-phase microemulsions are presented in Table 1. Because a Winsor IV microemulsion can be confused with a liquid crystal based only on the visual appearance, an additional analysis must be performed. The transparent one-phase systems were inspected using cross-polarized light microscopy to confirm whether they were Winsor IV microemulsions. Microemulsions are isotropic; thus, they do not interfere with polarized light [44]. A cross-polarized microscopy analysis confirmed the isotropic nature of all the six samples selected for the study.

Electrical conductivity is another fast and simple technique that offers valuable information about the microemulsion structure, especially regarding whether the continuous phase is an oily phase or an aqueous phase. Combined with other techniques, it helps to reveal the W IV microemulsion type, which can be oil in water (O/W), bicontinuous or water in oil (W/O). In general, a high conductivity value is characteristic of a bicontinuous microemulsion and especially an O/W microemulsion. Microemulsions with low conductivity values are classified as the W/O type [45]. The conductivity measurement results are depicted in Table 2. Using data from Table 1 and Table 2, it can be seen that the electrical conductivity increased as the amount of water in the samples increased. A sudden increase in electric conductivity has been interpreted in the literature as a percolation process [46]. Regarding the structural state of the microemulsion, it indicates a transition from a W/O microemulsion to a bicontinuous microemulsion to an O/W microemulsion [47]. Starting with sample F1.2 for which the electric conductivity suddenly increased to 18.37 mS/cm, it can be concluded that the formulations F1.3, F1.4 and F1.5 are O/W Winsor IV-type microemulsions. The variation in the conductivity with the water content suggests that samples F2.1 and F1.1 are O/W microemulsions, while F1.4 and F1.5 are O/W. It is presumable that F1.2 and F1.3 are in transitional stages, i.e., bicontinuous structures. The oil and water concentrations of these formulations and the DLS measurements also support this conclusion.

To obtain additional information about the Winsor IV microemulsion type, a measurement of the discontinuous-phase droplet size was performed using DLS, and the results are presented in Table 2. For each sample, the measurements were conducted in triplicate.

F1.2 and F1.3 exhibited high polydispersity (PdI values beyond 0.9); thus, these samples were not suitable for the DLS analysis, as it was expected that such concentrated systems could not be diluted for further measurements. For samples F2.1 and F1.1, W/O-type microemulsions, the main aqueous droplet diameter values were 16.67 ± 1.11 nm and 17.80 ± 1.96 nm, respectively, with no significant differences with an increase in the water content from 6.9% to 9.1%. In the opposite way, for samples F1.4 and F1.5, where an O/W structure was present, the size of the oil droplets was significantly higher at 80.76 ± 6.34 nm and 45.15 ± 3.65 for an oil content of 5.6% and 4.9%, respectively. It was noted that, for stabilization, samples F1.4 and F1.5 with O/W structures required a significantly lower amount of the surfactant mixture (50% and 43.9%) than the W/O microemulsions F2.1 and F1.2 (79.2% and 81.8%).

No significant changes (*p* > 0.05, *t* test) were observed regarding the size and size distribution of the liquid droplets in the selected microemulsions after 3 months of storage in the dark at 25 °C. For example, the main diameter initially recorded for the water droplets in sample F1.2 was 17.80 ± 1.96 nm, whereas after storage it was 14.35 ± 1.05 nm. For sample F1.5 with an O/W structure, the oil droplets showed initial value of 45.15 ± 3.65 nm; after storage, a value of 41.99 ± 2.36 nm was recorded.

**Figure 2:**
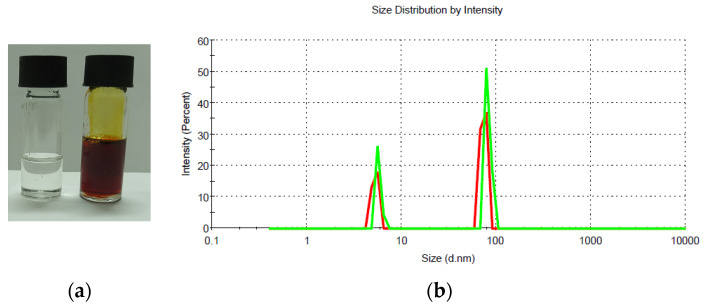
Visual appearance (**a**) and DLS diagram (**b**) of the O/W microemulsion F1.4 with and without encapsulated curcumin.

Microemulsion F1.4, which was considered the most favorable in terms of composition and structure for curcumin administration, was selected for further experiments. After drug encapsulation, the system remained perfectly clear and no separate phase was observed, as can be seen in Figure 2.

The encapsulation of curcumin in the optimized microemulsion F1.4 did not produce a significant increase in the size of the oil droplets (Figure 2) (from 80.86 nm ± 6.34 nm to 82.71 ± 7.19 nm) due to the entrapment of the drug molecules, as reported in other papers for microemulsions with a low oil content.

### 3.3. Preparation of the Gel Microemulsions

From the resulting Winsor IV microemulsions, two microemulsions were selected to be transformed into gel microemulsions. An O/W microemulsion with a reduced S_mix_ concentration but not the lowest oily phase concentration was selected, namely, microemulsion F1.4. From the same dilution line, a W/O microemulsion with the lowest aqueous-phase concentration was selected. As mentioned above, to increase the viscosity of the aqueous phase, NaH was used.

Sodium hyaluronate was selected as a thickening agent to produce gel microemulsions out of the polymeric derivatives currently used in cosmetic formulations, because the preliminary tests on the solubilization of the polymers in the microemulsions prepared in this study were unsuccessful. The addition of chitosan or diutan (another polymer tested) in a concentration ranging from 0.1 to 0.3% (*w*/*w*) to sample F1.4 led to the rapid formation of a solid separate phase regardless of the method used to incorporate the polymeric material. Diutan is a natural gum with a high molecular weight used as a gelator cosmetic ingredient, and it is produced via the aerobic fermentation of a bacterial strain, *Sphingomonas* sp., which contains units of L-rhamnose, D-glucose, D-glucuronic acid, and D-glucose in the backbone and L-rhamnose in the attached side chains. Its efficiency in thickening and biocompatibility are well recognized, but, unfortunately, its interaction with surfactants (both ionic and nonionic surfactants) often generates polymer–surfactant complexes with a rather low solubility [48]; thus, in the conditions of our experiments, obtaining a homogeneous gel microemulsion with diutan was not possible. The solubilization of chitosan was also investigated in the F1.4 sample, but the aqueous volume was too low to ensure a polymer concentration that sufficiently increased the viscosity of the microemulsion, and solid polymer–surfactant complexes were also present at concentrations beyond 0.1% (*w*/*w*). Thus, sodium hyaluronate was chosen as a thickening agent to modify the microemulsion viscosity.

Two concentrations of NaH were studied, as well as two different methods of polymer incorporation. The polymer concentration was calculated as the weight percentage of the aqueous microemulsion phase. In Table 3, photos of the results are displayed for both methods used for the NaH addition and the selected concentrations.

NaH added to the already prepared emulsion did not dissolve, and this is visible in the photos included in Table 3. For the F1.1 formulation, gel microemulsions were not obtained with any of the concentrations or methods. The second method (the preparation of a microemulsion using a polymer solution as the aqueous phase) proved to be the correct approach for the F1.4 formulation. Two gel microemulsions were further characterized and compared with the corresponding (parent) microemulsion to select one of the gel microemulsions for the loading capacity study.

### 3.4. Rheological Characterization of the Winsor IV Microemulsions and Gel Microemulsions

Another parameter that is influenced by the structural state of the microemulsion is rheology. However, rheological measurements on their own cannot be used to determine the microemulsion’s structure. Therefore, rheological measurements are used together with other techniques, such as electric conductivity, DLS and cross-polarized light microscopy, to establish the Winsor IV microemulsion type [49].

**Figure 3:**
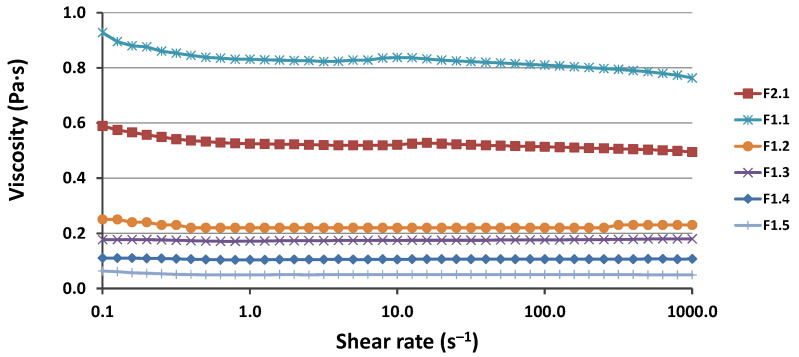
Viscosity as a function of shear rate for the obtained Winsor IV microemulsions.

According to the literature, at low-to-medium shear rates, bicontinuous microemulsions display a Newtonian behavior (constant viscosity), whereas shear thinning is found at high shear rates, most likely owing to bicontinuous structural fragmentation. Newtonian behavior is observed in discontinuous microemulsions over a larger range of shear rates [50]. As shown in Figure 3, even when the shear rate was significantly increased, the viscosity of the microemulsion remained nearly constant, with no indication of a rapid decrease in viscosity. Hence, the rheological measurements confirm the DLS and conductivity measurement results regarding the structure of the W IV microemulsion.

The Rheological data reflect the microemulsion composition (Table 1); therefore, as can be seen in Figure 3, the viscosity decreased as the water content increased. For all microemulsions, the viscosity was lower than 1 Pa·s. These results are in agreement with the data in the literature [51,52].

**Figure 4:**
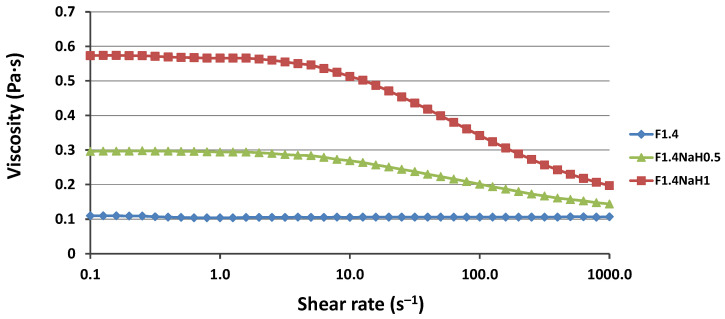
Viscosity as a function of shear rate for the F1.4 microemulsion and the gel microemulsions F1.4NaH0.5 and F1.4NaH1 derived from it.

As was expected and can be seen in Figure 4, by adding NaH to the microemulsion, the viscosity increased, forming gel microemulsions, which are easier to use for topical applications. Moreover, the viscosity increased significantly with an increase in the NaH concentration. When the F1.4 O/W microemulsions were thickened with NaH, the viscosity notably increased in the low-shear-stress zone. At the same time, the shear stress dependence in the flow curves increased. Therefore, when the shear rate exceeded 5 s^−1^, a shear-thinning phenomenon occurred. This effect was previously observed in other research papers, and it can be explained by the polymer molecules arranging according to the shear rate. At lower shear rates, polymer macromolecules are aggregated, but the aggregates are destroyed when a higher shear rate is applied [52,53].

### 3.5. Encapsulation Efficiency of Curcumin in Selected Delivery Systems

The curcumin encapsulation capacity of the F1.4 microemulsion and the corresponding gel microemulsion with 1% NaH, with the most increased viscosity, was evaluated and compared using quantification based on fluorescent spectroscopy. The resulting spectrum showed that the maximum emission of curcumin was about 525 nm (with the excitation wavelength set at 425 nm). The obtained values, according to the used parameters, were consistent with the literature data [35,54].

To establish the maximum quantity of curcumin that can be incorporated into the microemulsions and gel microemulsions, an excess of curcumin was added to the systems, followed by magnetic stirring and centrifugation to ensure that the samples were saturated and that no crystal of the active substance remained in the samples that were further analyzed. Using the data obtained from the calibration curve, the maximum curcumin quantity that could be incorporated into the selected samples was established. The maximum amount of curcumin that could be incorporated into F1.4 was 3.01 ± 0.12 mg/mL, and in F1.4NaH1 it was 3.35 ± 0.12 mg/mL. The gel microemulsion with hyaluronate showed a slightly higher efficiency for the encapsulation of curcumin, probably due to the possibility of establishing an interaction between the polymer chain and the drug molecules at the interface. Compared to the microemulsions previously obtained by the same research group [24], using a vegetal oily phase and Tween 80 and Plurol^®^ Diisostearique CG as surfactants, the encapsulation efficiency of the F1.4 systems was lower. Since curcumin possess a very poor water solubility, the lower incorporation capacity can be explained by the increased water quantity in F1.4 and the decreased oil and S_mix_ quantity compared to those of the previously reported microemulsions. However, the amount of curcumin incorporated into formulations F1.4 and F1.4NaH1 is comparable with the data reported for other microemulsions with a low oil content [26,55,56].

### 3.6. FTIR Analysis of the Winsor IV Microemulsions and Gel Microemulsions

**Figure 5:**
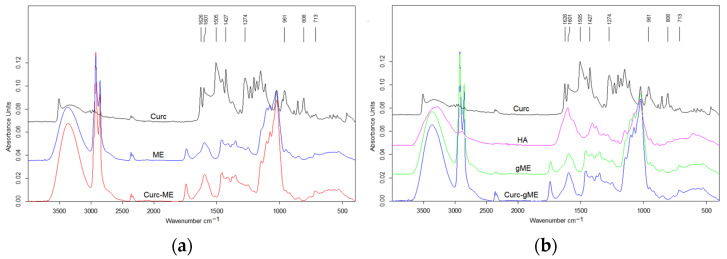
The FTIR spectra of curcumin encapsulated in microemulsion (**a**) and encapsulated in gel microemulsion (**b**), compared to void carriers and curcumin powder.

Possible interactions of the encapsulated curcumin with the microemulsion and gel microemulsion used as vehicle were investigated using FTIR analysis. The spectra of pure drug and the drug-loaded formulations are presented in Figure 5.

The spectra obtained for curcumin powder showed characteristic peaks for the crystalline drug, in accordance with other data in the literature [57]. The signal in the region of 3500 cm^−1^ is related to the phenolic O−H group, the peak at 1506 cm^−1^ corresponds to the aromatic C−C stretching vibration and the one at 1274 cm^−1^ is related to aromatic ether groups. The peaks at 1626 and 1601 cm^−1^ can be assigned to C=O and C=C vibrations and symmetric aromatic ring (C=C) stretching vibrations. The peaks at 961, 808 and 961 cm^−1^ can be associated with the bending vibration of the C–H alkene group.

Due to the presence of similar functional groups in the chemical structure of the microemulsion components (Plantacare and Akypo surfactants, and capryl/caprylate triglyceride, respectively), the peaks in the FTIR spectrum of the microemulsion contain overlapping signals, mainly coming from O-H group vibration (broad peak in the region between 3600 and 3200 cm^−1^), and from H-O-H bending at 1594 cm^−1^. Other peaks at 2922 and 2854 cm^−1^, corresponding to stretching vibrations of the CH_2_ groups and the C-O-C stretch corresponding peak at 1024 cm^−1^ were also present, consistent with reports in the literature [58]. In the spectra of the drug-loaded microemulsion (Figure 5a), the characteristic peaks of curcumin were hindered by the signals of the carrier components, due to the low content of the active compound. The same situation was found in the spectra of curcumin encapsulated in the gel microemulsion (Figure 5b). However, in the spectra of void carriers (microemulsion and gel microemulsion) compared to the curcumin-encapsulating ones, no changes in the position or intensity of the characteristic peaks were noted; thus, it is presumable that drug loading does not induce chemical interactions or significant alterations in the carrier structures.

### 3.7. In Vitro Antioxidant Activity

Curcumin is presently proposed as an active ingredient in skincare products due to its antioxidant properties, as it has been proven to act as a potent free radical scavenger. Thus, preserving the antioxidant activity of CURC when it is encapsulated in various formulations is a crucial task when developing a novel drug delivery system.

The stability of the antioxidant activity of curcumin encapsulated in the O/W microemulsion (the F1.4 sample) compared to that of the corresponding gel microemulsion with hyaluronate (the F1.4NH1 sample) was evaluated based on the capacity to scavenge DPPH free radicals. Because antioxidant efficiency is concentration dependent, it is expressed as IC_50_, the sample concentration that inhibits 50% of free radicals (50% reduction of DPPH). The value of 14.26 ± 0.60 μg/mL obtained for the CURC encapsulated in the microemulsion was slightly lower than the value obtained for the standard solution of CURC dissolved in ethanol (12.36 ± 0.39 μg/mL), suggesting that the interaction between the drug molecules and the oil and surfactant mixture in the microemulsions led to a decrease in the amount of CURC available to react with DPPH free radicals. The presence of hyaluronate in the gel microemulsion (sample F1.4NH1) further decreased the antioxidant activity to 17.32 ± 0.41 μg/mL. A decrease in the antioxidant activity of curcumin due to entrapment in nanocarriers was also reported in the literature.

Despite the reduction in the antioxidant activity of curcumin due to interactions with the constituents of the microemulsion, the antioxidant efficiency of the studied colloidal systems was very high because of the possibility of solubilizing increased amounts of drug compared to aqueous or alcoholic solutions. Thus, the proposed microemulsion-based vehicles, proven to have a superior solubilization capacity and good antioxidant properties, are a valuable solution for skin-protective formulations.

### 3.8. In Vitro Curcumin Release from Microemulsion-Based Formulations

The release profile of the drug from the studied formulations are important in order to select the best carrier to ensure an adequate delivery rate.

**Figure 6:**
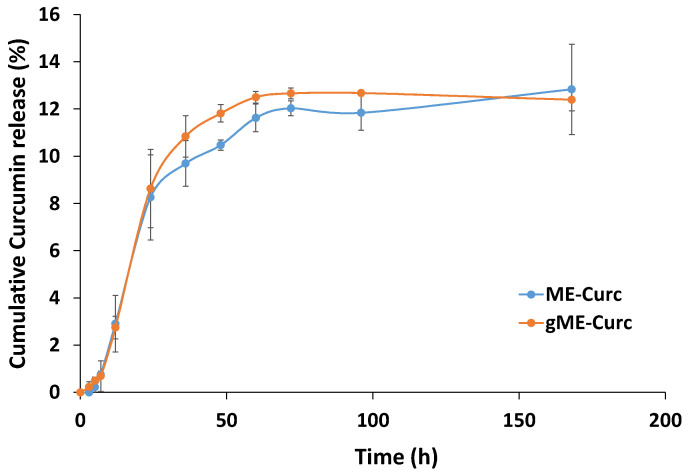
Release kinetics of curcumin encapsulated in an microemulsion (sample F1.4) and the related gel microemulsion (sample F1.4NaH1).

The curves for the cumulative amount of curcumin released from the selected carriers (microemulsion F1.4 and gel microemulsion F1.4NaH1) are shown in Figure 6.

For both systems, an accelerated release up to 24 h was observed, followed by a constant release until 60 h. A lag time of 3 h is observed for the drug release from microemulsion, while in the case of gel microemulsion, the lag time seemed to be shorter. After 60 h, there was a flattening of the release curve up to 168 h. The release profile is comparable with results from other literature reports [59], but the curcumin in the present nanosystems was released at a slower rate, making it feasible to attain a sustained release. In cosmetic applications where a slow and extended release of active ingredients is pursued, this property is crucial. On the segment from 48 h to 72 h for the gel microemulsion, the released amount was higher (*p* < 0.05); on other segments, the differences in the released amounts were not statistically significant. Additionally, it was shown that the release profile of curcumin was almost unaffected by increasing the viscosity of the microemulsions, namely the gel microemulsion. Both systems showed similar release profiles; therefore, the results support the data obtained from the permeation study (see Section 3.9).

The release data of curcumin from microemulsion-based carriers were fitted using mathematical models used in the literature, with following equations:First-order model: (100 − Q_t_) = exp (k_1_ × t)Higuchi model: Q_t_ = k_H_ × t^1/2^Korsmeyer–Peppas model: M_t_/M_∞_ = k_KP_ × t^n^Peppas–Sahlin: M_t_/M_∞_ = k_1_ × t^m^ + k_2_ × t^2m^Weibull: M_t_ = M_max_ × [1 − exp (−t^β/α^)]
where Q_t_ represents the cumulative drug released at a particular time t, and M_t_ and M_∞_ are the released drug at time t and the maximum amount of released drug (at infinite time). The constants k_0_, k_1_, k_H_ and k_KP_ are the release constants defined in the different models. Parameter n is a diffusional exponent in Korsmeyers–Peppas model. The values of the Korsmeyers–Peppas n parameter indicates the release mechanism (n ≤ 0.43 corresponds to a Fickian diffusion, n ≥ 0.85 corresponds to a relaxational transport and intermediate n values between 0.43 and 0.85 correspond to a combined diffusion and relaxation release process).

The Peppas–Sahlin model is derived from the Korsmeyer–Peppas model, and introduces separate relative contributions of diffusional and relaxational terms for the drug release mechanisms [60]. The coefficient m is a Fickian diffusional exponent, depending on the shape of the formulation release curve.

As an empirical model, without correlations with the release mechanism, the Weibull kinetic model was used, in which the term β is a parameter that describes the shape of the curve progression, while α is a scale parameter associated with the time scale of the release process.

Zero-order and Hixon–Crowell models were also tested, but the low values of the correlation parameters led to the exclusion of these models from further analysis.

The goodness of fit was evaluated using the adjusted correlation coefficient (R^2^_adj_), the Akaike information criterion (AIC) and the model selection criterion (MSC), in order to find the more suitable model.

**Figure 7:**
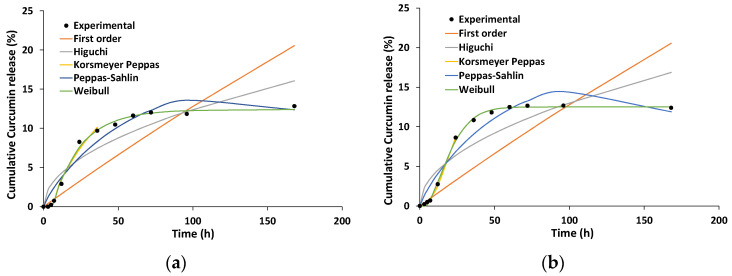
The theoretical kinetic profiles of curcumin from microemulsion (**a**) and gel microemulsion (**b**) from various release models.

The main parameters of each model are shown in Table 4 and the generated theoretical curves are presented in Figure 7.

When comparing different models, the highest value of R^2^_adj_ and lowest value for AIC indicate the best fitting. Analysis of the statistic parameters from Table 4 lead to the conclusion that in both cases (curcumin loaded in microemulsion and curcumin loaded in gel microemulsion), the release kinetics was best described by the Korsmeyer–Peppas and Weibull models. For both formulations, the Weibull model produced a relatively good prediction for the maximum release and the lag time. Although the parameters β and α in the Weibull model are non-physical in nature [61], recent studies demonstrated close correlations between their value and the release mechanism [62]. As it is shown in Table 4, for the release profile of curcumin from the microemulsion, the value of the β parameter was in the range of 0.75–1.0, which indicates a combined mechanism. In the case of the release from the gel microemulsion, the parameter β > 1, which is associated with a more complex mechanism of drug release. Such a situation where the β value is higher than 1 was previously reported for the release from a lyotropic liquid crystal formulation, with a high content of surfactant and polymer in its composition [63].

Analyzing the results from the Korsmeyer–Peppas model, one can observe that for the drug release from both formulations, the diffusional parameter n was higher than 0.43, with a value of 0.617 for the microemulsion and >0.85 for the gel microemulsion. Thus, it is presumable that for the microemulsion, the release mechanism is a non-Fickian process, while for the gel microemulsion, it is a super case II. Similar information could be obtained from the Peppas–Sahlin model, where k_1_ is related to the diffusional contribution and k_2_ to the relaxational one. The k_1_ values were similar for the curcumin release from both microemulsion-based carriers, and very close values were also obtained for k_2_. The values obtained for k_2_ indicate the inhibition of the release, which is consistent with the curve shape, showing a constant release after approximately 24 h, similar to other drug release kinetics from lipid and polymeric matrices [64].

### 3.9. In Vitro Permeation Study

To investigate the skin transfer efficiency of curcumin, as a model cosmetic active ingredient, in the designed microemulsion and gel microemulsion, an in vitro permeation study was performed. The experiments were conducted using a Franz diffusion cell with a membrane, Strat-M^®^, as a replacement for animal/human skin. This material, due to its complex multilayer structure, has been documented to possess a structure similar to that of human skin, and it is considered to be an affordable, ethical and reliable alternative to the use of animal or human skin for the testing and optimization of pharmaceutical formulations [42].

**Figure 8:**
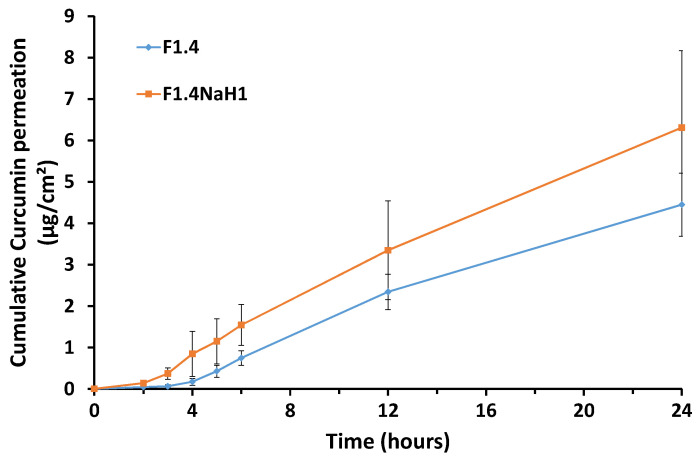
In vitro permeation of curcumin from microemulsion and gel microemulsion through the Strat-M^®^ membrane.

**Figure 9:**
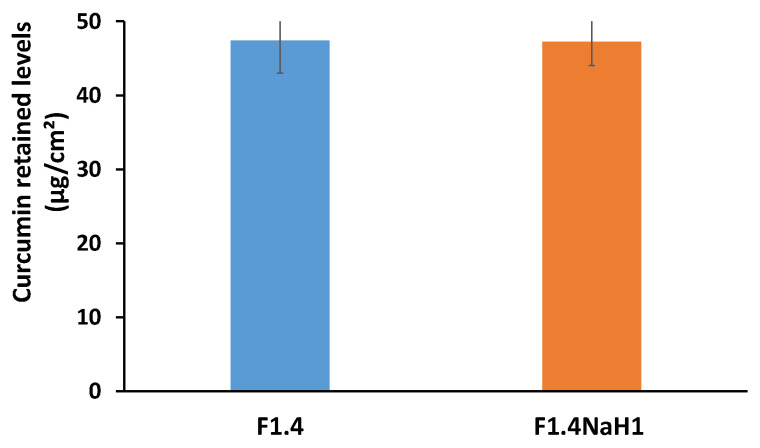
Total curcumin retained in the Strat-M^®^ membrane after 24 h.

The results of the permeation kinetics and the total retention in the membrane are presented in Figure 8 and Figure 9, respectively.

The cumulative permeated quantity of curcumin at 24 h was not influenced by the presence of the polymeric thickening agent (*p* > 0.05), as is shown in Figure 9, and the release profile of curcumin was similar from the microemulsion (sample F1.4) and from the gel microemulsion (F1.4NaH1) (Figure 8). For both the microemulsion and gel microemulsion carriers, an average lag time of 3 h was noted, a period in which the constituents of the vehicle were embedded into the membrane layers and allowed the diffusion of the drug molecules.

The values obtained for the steady-state fluxes and the apparent permeability coefficients of the selected microemulsion and gel microemulsion loaded with curcumin are displayed in Table 5, and they are comparable with the data in the literature [43].

Comparing the values in Figure 4 and Figure 5, it can be concluded that the amount of curcumin that was retained in the Strat-M^®^ membrane was higher than the amount that permeated through the membrane. The addition of the gelling agent to the aqueous phase did not have a notable impact (*p* > 0.05) on the levels of curcumin retained in the model membrane. For cosmetic products developed for anti-aging skin treatment, the targets for efficient delivery are the dermis and the epidermis. Thus, the ability of the proposed microemulsions to retain the encapsulated curcumin in the model membrane suggests that these microemulsions could be suitable nanostructured carriers for the enhancement of this drug’s antioxidant effects on the targeted skin layers. The lipophilic nature of CURC, the small dimensions of the oil droplets and the rather high content of surfactants compete synergistically to concentrate drug molecules into the model membrane. However, the preferential penetration of the stratum corneum of curcumin encapsulated in a microemulsion, compared to a commercial pharmaceutical base cream, was reported after an in vivo study on human volunteers [65]. Unfortunately, in this mentioned study, the microemulsion used was prepared with ingredients such as Poloxamer 331 (Synperonic PE/L 101) and Pelemol BIP (a eutectic mixture of N-butylphthalimide and N-isopropyl-phthalimide), which are unusual for cosmetic products.

### 3.10. In Vitro Cytotoxicity and Cellular Internalization

The toxicity of the empty and curcumin-loaded gel microemulsion formulations (sample F1.4H1) was investigated using fibroblast cells, as a model of normal human cells present in many organs.

**Figure 10:**
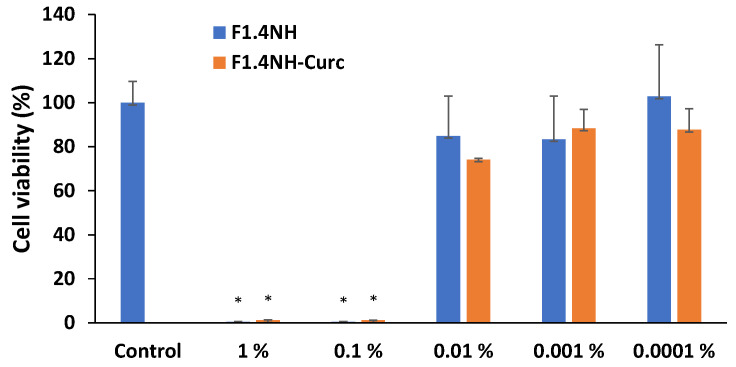
Cellular viability after 24 h exposure to various concentrations of empty and CURC-loaded gel microemulsion formulations. (*) *p* < 0.05.

In Figure 10, the results of cellular viability based on the MTT test are shown.

As expected for very concentrated formulations with a high content of surfactants and ethanol as a co-solvent, the gel microemulsion exhibited a drastic reduction in cell viability at a concentration above 0.01% (X10 dilution of the original samples). The viability showed a sharp increase at lower concentrations (dilutions higher than X100), with a viability with no significant difference from that of the untreated cells. Both the empty gel microemulsion F1.4 and the one with encapsulated CURC (at a concentration of 3.30 mg/mL, close to maximum drug solubility) showed similar variations in toxicity, suggesting that the main cytotoxic effect is due to the presence of the drug delivery system itself.

**Figure 11:**
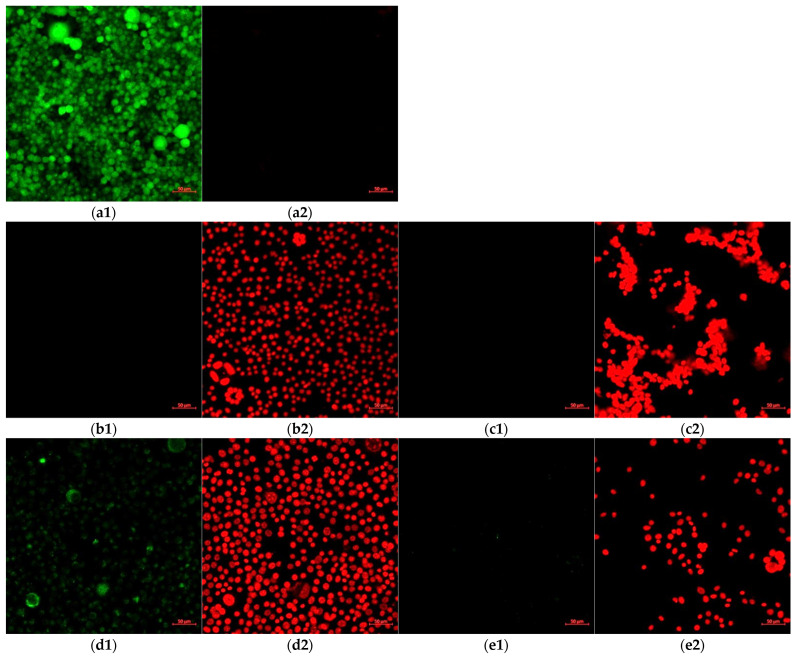

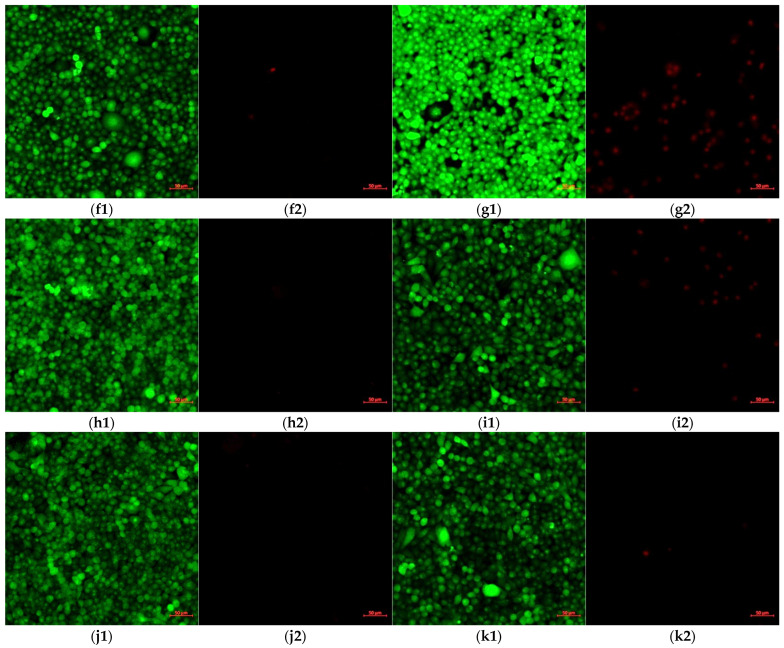
Confocal microscopy images of fibroblast cultures incubated with various concentrations of CURC-loaded F1.4NH1 samples compared to those incubated with gel microemulsion F1.4NH1 without drug. Images (**a1**,**a2**) are from control cultured cells without treatment. Rows 2–5 correspond to the decimal dilutions of the initial concentration (1%) of the treatment as follows: (**b1**,**b2**) and (**c1**,**c2**) initial concentration; (**d1**,**d2**) and (**e1**,**e2**) dilution 1/10; (**f1**,**f2**) and (**g1**,**g2**) dilution 1/100; (**h1**,**h2**) and (**i1**,**i2**) dilution 1/1000; (**j1**,**j2**) and (**k1**,**k2**) dilution 1/10,000. The curcumin content in the F1.NH1 gel microemulsion was 3.30 mg/mL. Panels denoted with 1 are samples stained with calcein, while those denoted with 2 are samples stained with PI.

In order to perform an in-depth evaluation of the influence of the carrier on the toxicity against fibroblasts, confocal images were analyzed. The confocal images of the cell cultures after treatment with various concentrations of the CURC-loaded carriers are presented in Figure 11.

The dose-dependent cytotoxicity of the gel microemulsion proposed in this study was highlighted by the LIVE/DEAD test using a decimal dilution series (Figure 11). It is noteworthy that, in the experimental conditions, in the cells incubated with the vehicle without the drug at a 1/100 dilution, propidium iodide occupied the entire cytoplasm with the destabilization of the nuclear envelope (determined through the size differences compared to the nuclei of the dead cells after further dilution). For the cells incubated with the CURC-loaded gel microemulsion at the highest concentration, the nuclei of dead cells were identified, but there were also dead cells in which the nuclear envelope was destabilized. The differences between the empty gel microemulsion and the CURC-loaded carrier suggest a protective effect of curcumin in stabilizing the nuclear envelope.

At higher concentrations, both the empty and drug-loaded nanosystems had a high toxicity level, as indicated by the presence of cells with red nuclei and cells in which the fluorophore propidium iodide was found throughout the cytoplasm, which suggests the destabilization of the nuclear envelope in certain cells. In the cultures treated with the empty gel microemulsion, all cells were dead, whereas for the cells treated with a CURC encapsulating system, the fluorescence of calcein superimposed on the fluorescence of propidium iodide was highlighted in places, which suggests a protective effect on the viability of curcumin compared to the carrier without the drug.

In the experimental conditions used in this study, after further dilutions of the gel microemulsions, the cell viability was similar to that of the control without treatment, without fluorescence anomalies.

The high level of toxicity expressed by the gel microemulsion at moderate and high concentrations is explained by the elevated content of surfactant in the formulation, together with the presence of ethanol as a co-solvent. Another reason is also the prolonged period of incubation with the treatment, as most of the reported data were obtained after 1–5 h of incubation. These experimental conditions were chosen in order to mimic the conditions of using a regular anti-aging cosmetic cream, which is usually developed to be applied to and maintained on the skin for at least 8–12 h, and to also evaluate the effect of an incorrect skin cleaning procedure for make-up removal.

**Figure 12:**
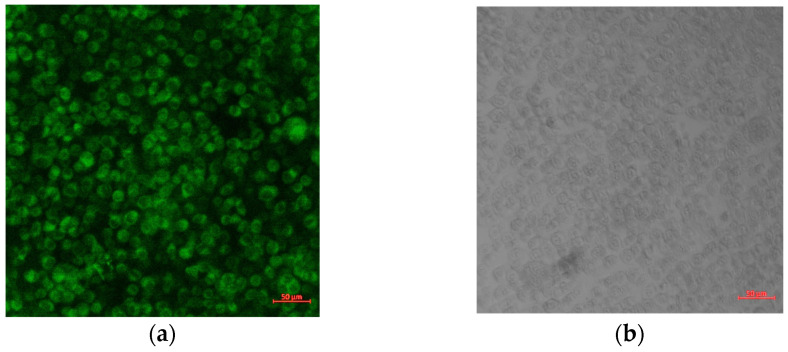
Confocal images of self-fluorescence of CURC (**a**) compared with transmission microscopy (**b**) on cells incubated with CURC-loaded gel microemulsion at 1/1000 dilution.

In order to investigate CURC cellular internalization, a microscopic evaluation of its autofluorescence was performed using laser excitation at 458 nm, and an evaluation was also performed at 470–570 nm (without the use of other fluorophores) (Figure 12).

**Figure 13:**
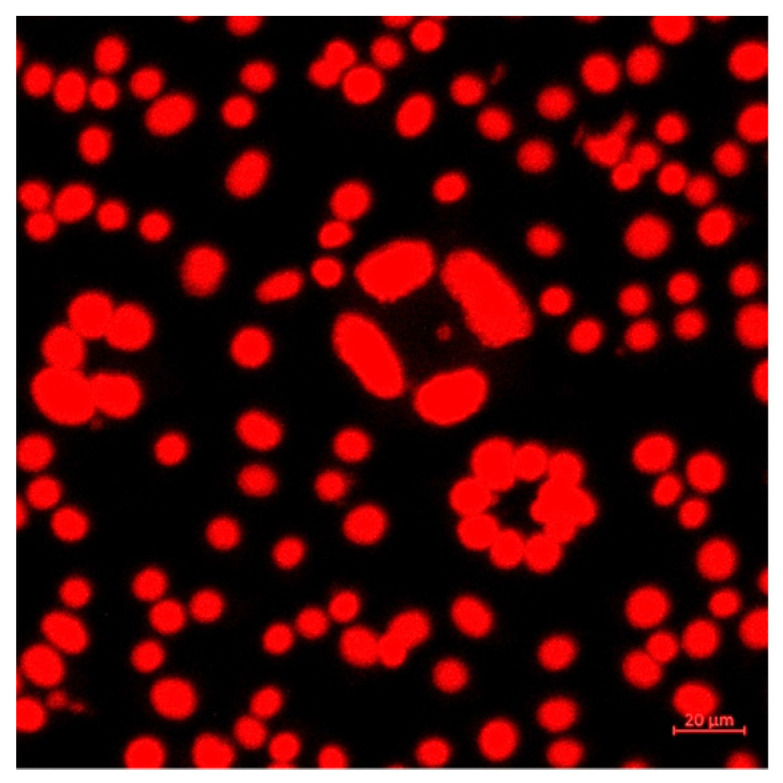
Magnified image of fibroblasts incubated with CURC-loaded sample (dilution 1/100) and stained with propidium iodide (LIVE/DEAD test).

In Figure 13, a detailed image of a cell culture incubated with the drug-loaded gel microemulsion is shown to further elucidate the protective influence of CURC and the interaction with the cell membrane.

The existence of circular cellular associations highlighted in the conditions of exposure to high doses of microemulsions, enlarged in Figure 13, was not present in the control conditions. This suggests the initial co-existence of cell viability with cell division accompanied by a lack of cell mobility, even if the fluorescence was recorded using the propidium iodide channel. The correlation between the predominant disposingt of Curcumin in the proximity of the membranes (Figure 12) and the existence of circular associations in the conditions of high concentrations (Figure 11(b1–c2)) suggests that these toxic effects are due to the carrier components and that curcumin has a protective effect. The decrease in cytotoxicity is probably governed by the interactions between the transmembrane anchoring proteins to the substrate and the cytoskeleton protected by the presence of CURC, particularly by preserving the arrangement and function of integrins.

## 4. Conclusions

The objective of this study was to develop a novel microemulsion-based transdermal formulation to be used as a curcumin drug delivery system for cosmetic use. Finding a non-toxic surfactant mixture to form a microemulsion was the challenge. Alkylpolyglucoside (Plantacare) was selected as the primary surfactant because it is approved for use in cosmetic formulations due to its naturality index and low toxicity. Plantacare combined with Akypo as a cosurfactant enables single-phase microemulsions to be obtained with relatively low surfactant mixture concentrations. The fluidization and flexibility of the S_mix_ film were made possible by the Akypo cosurfactant due to the linker in the structure. In this study, the phase behavior of the water–caprylic/capric triglycerides (CCTs)–Plantacare–Akypo system was investigated for the first time, and the results in the formulation of a Winsor IV microemulsion were reported. An extended area of microemulsion was obtained at low surfactant mixture concentrations. Several representative single-phase microemulsions were obtained and characterized. The O/W-type Winsor IV microemulsion and the gel microemulsion derived from it using hyaluronic acid salt as a thickening agent showed a high capacity for the encapsulation of curcumin (more than 3 mg/mL), compared to CURC solubility in water or ethanolic solutions. The cytotoxicity of the CURC-loaded optimized gel microemulsion was due to the formulation components; in this study, cytotoxicity was particularly observed at high concentrations and after a very long period of treatment (48 h of incubation). A protective effect of the encapsulated CURC was noticeable against the damaging effect of the carrier itself, even with treatments using high concentrations. Dilution of the gel microemulsion samples (1/100) to which the cells were exposed led to a cellular viability similar to that of the control.

An in vitro permeation study indicated a gradual and controlled release, so the developed systems ensure a prolonged effect of the treatment. The CURC encapsulated in both the microemulsion and gel microemulsion was preferentially retained in the model membrane (mimicking the skin), at an amount that was larger than the amount that permeated through it. The proposed gel microemulsion, with a low content of surfactants, exhibited an increased drug loading efficiency and high permeation through a model membrane, and thus could be considered as a promising carrier for topical application of curcumin in anti-aging cosmetic products.

## Figures and Tables

**Table 1:** Weight compositions of W IV microemulsions.

Sample Name	Concentration % (*w*/*w*)		
	Water	CCT	S_mix_
F2.1	6.9	13.9	79.2
F1.1	9.1	9.1	81.8
F1.2	24.5	7.6	67.9
F1.3	38.5	6.2	55.3
F1.4	44.4	5.6	50.0
F1.5	51.2	4.9	43.9

**Table 2:** Physical parameters of electrical conductivity, polarized microscopy and dynamic light scattering for the Winsor IV microemulsions.

ME Formulation Name	Water Concentration (% wt.)	Electric Conductivity (mS/cm)	D (nm)	Polarized Microscopy
F2.1	6.9	2.44	16.67 ± 1.11	Isotropic (ME)
F1.1	9.1	3.40	17.80 ± 1.96	Isotropic (ME)
F1.2	24.5	18.37	n.a	Isotropic (ME)
F1.3	38.5	41.10	n.a	Isotropic (ME)
F1.4	44.4	46.50	80.76 ± 6.34	Isotropic (ME)
F1.5	51.2	53.70	45.15 ± 3.65	Isotropic (ME)

**Table 3:** Visual appearance of the F1.1 and F1.4 samples after addition of the polymer.

ME Formulation Name	NaH 1% Method 1 (% wt.)	NaH 1% Method 2 (% wt.)	NaH 0.5% Method 2 (% wt.)
F1.1	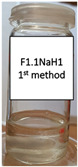	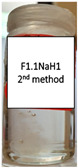	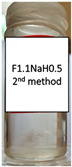
F1.4	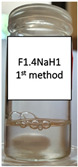	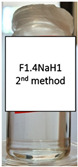	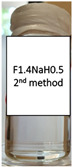

**Table 4:** Parameters of drug release models obtained for curcumin encapsulated in microemulsion and gel microemulsion.

**Model**								
**First Order**			**Higuchi**			**Korsmeyer–Peppas**		
**Parameter**	**ME**	**gME**	**Parameter**	**ME**	**gME**	**Parameter**	**ME**	**gME**
k_1_	0.001	0.001	k_H_	1.239	1.301	k_KP_	1.260	0.031
						n	0.617	1.7742
						T_lag_	6.6	–
R^2^_adj_	0.5446	0.4562	R^2^_adj_	0.8463	0.8015	R^2^_adj_	0.9776	0.9970
AIC	61.9226	65.2946	AIC	48.8859	53.2009	AIC	8.9946	−8.2822
MSC	0.2931	0.2895	MSC	1.3794	1.2973	MSC	3.1422	5.3132
**Peppas–Sahlin**			**Weibull**					
**Parameter**	**ME**	**gME**	**Parameter**	**ME**	**gME**			
k_1_	0.493	0.522	β	0.9475	1.494			
k_2_	−0.004	−0.005	α	16.1633	81.972			
			M_max_	12.41	11.94			
			T_lag_	6.25	3.95			
R^2^_adj_	0.9353	0.9319	R^2^_adj_	0.9940	0.9976			
AIC	40.0999	41.9615	AIC	12.0733	2.1714			
MSC	2.1116	2.2339	MSC	4.4471	3.6953			

**Table 5:** Parameters of the in vitro permeability study: steady-state fluxes and apparent permeability coefficients of selected microemulsion and gel microemulsion loaded with curcumin.

Sample	Time (h)	J_s_ (µg/cm^2^∙h)	P_ap_ (10^−3^ cm/h)
F1.4	4	0.17	0.055
	5	0.26	0.088
	6	0.32	0.107
	12	0.27	0.089
	24	0.18	0.059
F1.4NaH1	4	0.85	0.284
	5	0.31	0.102
	6	0.40	0.132
	12	0.30	0.101
	24	0.25	0.083

## Data Availability

Not applicable.
